# Spatial Dependence of Physical Attributes and Mechanical Properties of Ultisol in a Sugarcane Field

**DOI:** 10.1155/2015/531231

**Published:** 2015-06-17

**Authors:** Uilka Elisa Tavares, Mário Monteiro Rolim, Veronildo Souza de Oliveira, Elvira Maria Regis Pedrosa, Glécio Machado Siqueira, Adriana Guedes Magalhães

**Affiliations:** ^1^Departamento de Engenharia Agrícola, Universidade Federal Rural de Pernambuco (UFRPE), Rua Dom Manoel de Medeiros, s/n, Dois Irmãos, 52171-900 Recife, PE, Brazil; ^2^Centro de Ciências Agrárias e Ambientais, Universidade Federal do Maranhão, BR-222, KM 04, s/n, Boa Vista, 65500-000 Chapadinha, MA, Brazil

## Abstract

This study investigates the effect of conventional tillage and application of the monoculture of sugar cane on soil health. Variables like density, moisture, texture, consistency limits, and preconsolidation stress were taken as indicators of soil quality. The measurements were made at a 120 × 120 m field cropped with sugar cane under conventional tillage. The objective of this work was to characterize the soil and to study the spatial dependence of the physical and mechanical attributes. Then, undisturbed soil samples were collected to measure bulk density, moisture content and preconsolidation stress and disturbed soil samples for classification of soil texture, and consistency limits. The soil texture indicated that soil can be characterized as sandy clay soil and a sandy clay loam soil, and the consistency limits indicated that the soil presents an inorganic low plasticity clay. The preconsolidation tests tillage in soil moisture content around 19% should be avoided or should be chosen a management of soil with lighter vehicles in this moisture content, to avoid risk of compaction. Using geostatistical techniques mapping was possible to identify areas of greatest conservation soil and greater disturbance of the ground.

## 1. Introduction

In the State of Pernambuco, Brazil, the sugarcane agroindustry occupies a large agricultural area, contributing to the significant production of sugar and alcohol and providing social value by generating direct jobs in rural and industrial areas [1].

According to Goldemberg et al. [2] and Gomes et al. [3], the coastal tablelands in the Northeast region in Brazil are exploited for agriculture and have significant economic value. In this region, some of the soil can be classified as cohesive owing to climatic factors and relief favoring the formation of this kind of horizon. The cohesive soils have a hard to very hard consistency when dry and are friable when moist [4]. They have high landscape spatial variability, which interferes directly with the production of cane sugar.

Conventional tillage can be defined as the revolving of topsoil to provide a favorable environment for the growth of crops and contribute to increased productivity and reduced costs through soil decompaction; incorporation of lime and fertilizer; increases in pore spaces, permeability, and the storage of air and water; and weed control [5–8]. Agricultural machinery and implements are used to maximize production and income but used indiscriminately can cause compaction and the deterioration of soil quality, fertility, and productive capacity over a long time [7, 9].

Increased soil compaction also increases the preconsolidation and decreases compression stress, causing adverse conditions, like decrease in soil permeability and macroporosity, for the growth of crops [10]. The continuous cultivation of sugar cane, for many years, can significantly increase microporosity, and the increase in the subsurface layers is due to the intensive use of machinery promoting compression and pore clogging by clay illuviation [11].

When soils receive a load due to action for agricultural machinery and equipment, they undergo deformation and compaction whose intensities are related to their carrying capacity [6]. The laboratory test that can simulate the passage of the tractor on the ground is the compressibility test, and through it preconsolidation tension and compression ratio are obtained [6]. The preconsolidation tension (*σp*) expresses the maximum load that the soil has undergone throughout its history, indicating whether the ground has been under tension due to the passage of an agricultural vehicle or suffered an elastic deformation (recoverable) or plastic (nonrecoverable) [6].

**Figure 1:**
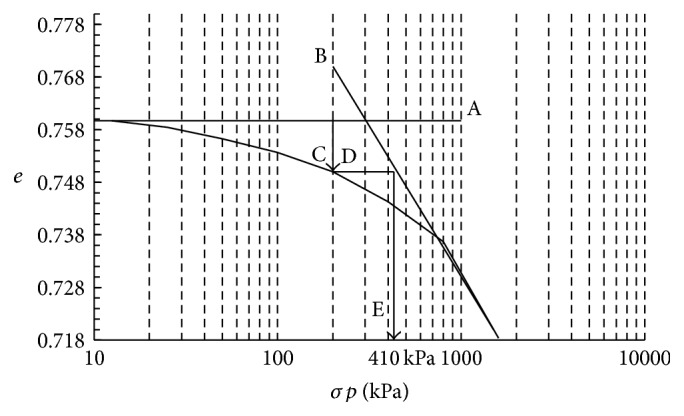
The Pacheco & Silva method to obtain the preconsolidation stress.

The preconsolidation tension is obtained from the relationship between the logarithm of the vertical tension applied to soil and parameters related to the structure of the soil such as voids, porosity, or bulk density [6]. This relation plotted a curve that has two distinct regions: the first, where small elastic and reversible deformations occur, Identified in the curve of figure graph 1, it means that while the tractor present a load within this graph range, the traffic will not cause additional compression; other regions correspond to the straight virgin compression (highlighted by the line B of Figure 1) from which the tension applied to the soil leads to irreversible deformations. The value of preconsolidation tension separates these regions [6].

Studying the stress strain consolidation of soil is important because the agricultural production system has been enhanced through the use of increasingly powerful and heavy farm machinery [6]. Lamande et al. [13] found that subsoil compaction can persist for 30 years after a single pass of a combined heavy machinery tractor and trailer.

Determining the spatial variability of physical attributes directly or indirectly related to the soil, such as density, moisture content, and soil texture, allows better control of crop production and environmental monitoring [4, 14]. Rabbi et al. [15] selected, through geostatistics techniques, soil quality index maps using a combination of physical attributes, such as bulk density, sand, silt, and clay, that can be used to evaluate the spatial variability of soil physical quality in an agricultural field.

The objectives of this work were to evaluate the spatial dependence structure of physical and mechanical attributes of soil and spatial correlation between these variables and to investigate the effect of soil wetting in their preconsolidation stress of the Ultisol at a depth of 0.0–0.20 m in a sugarcane field, giving direction to define a practical way of soil health maintenance.

## 2. Material and Methods

### 2.1. Study Area

The Ultisol (Table 1) was used for growing the sugarcane variety RB 92579, from Usina Santa Teresa in the Northern Forest region of Pernambuco, Brazil. The coordinates are 7°33′38′′ south latitude and 35°00′09′′ west longitude. The rainfall at the collection site was 1521.5 mm and the sugarcane was in its third harvest. The area received irrigation with vinasse, a residual substance left after sugarcane alcohol distillation, and soil tillage was conducted with Subsoilers and Disk Harrows implements.

### 2.2. Soil Sampling

The collections were made between May and November 2010 on days of lower rainfall. The cutting of sugarcane in the chosen area occurred in December 2010. Forty-nine soil samples were collected with disturbed soil to measure the contents of sand, silt, clay, consistency limits (liquid limit and plasticity index) and moisture content; and with undisturbed soil to measure bulk density and preconsolidation stress. The soil samples were collected in a square grid (20 m × 20 m by distance) composed of 7 rows and 7 columns at 0.00–0.20 m in depth. The total area was of 120 m × 120 m.

### 2.3. Soil Mechanics and Physical Analysis

The bulk density (BD) was obtained from undisturbed soil samples collected with the aid of Uhland sampler and a volumetric ring 0.05 m high and 0.02 m in diameter, according to the methodology of EMBRAPA [16]. In order to represent the BD in depth of 0.00 to 0.20 m, soil samples were collected with the Uhland sampler depths between 7.5 and 12.5 cm, which corresponds to the central point of the profile. The gravimetric moisture content (*θ*) was obtained as the relation between the mass of water in the soil and the dry mass of soil [16].

The texture was determined by a Bouyoucos densitometer with a dispersion of 50 g of air-dried soil in 25 mL of sodium hexametaphosphate buffered with sodium carbonate. The sand was separated by sieving, the clay by sedimentation, and the silt by subtracting the first two values from the total [16].

The liquid limit (LL) and plastic limit (PL) were determined according to ABNT [17] and ABNT [18], respectively, and the plasticity index (PI) was obtained from their difference.

Soil compressibility was evaluated in samples with a preserved structure collected in metal rings 0.025 m high and 0.065 m in diameter from a depth of 0.00–0.20 m, according to NBR 12007/90 [19]. A spatula was used to remove excess soil from the upper and lower surface of the ring filled with the soil. The whole mass of soil over the ring odometer was saturated by capillarity for 24 h and then subjected to pressures corresponding to the water content corresponding to field capacity (CC) and the permanent wilting point (PWP) observed by Bebé et al. [1] for the Ultisol in the experimental area: 0.1 and 0.19 kgkg^−1^, respectively. During the test, loads were applied at 12.5, 25, 50, 100, 200, 400, 800, and 1600 kPa, with adaptations for unsaturated soil, with a 30 sec duration of load application. Some authors studied and discussed the compressive behavior soil, applying compressive stress for different loading times, that is, 0.5 sec or 2 min, to resemble the short application of load that occurs in the field [20, 21]. Salire et al. [22] used a loading time of 2 min for each stress.

The software Compress 1.0 was used for the construction of curves determining the compressibility and tension for preconsolidation (*σp*). This parameter is defined in the void ratio (*e*) and log tension (*σ*) graph. The Pacheco & Silva method was used to determine the preconsolidation tension [19]. This method considers the meeting point of the horizontal line A with line B (also known as virgin compression line) and arrows C, D, and E are the steps needed to define the preconsolidation tension value (Figure 1).

### 2.4. Statistical and Geostatistical Analyses

The variables were analyzed using the descriptive statistics of the mean, median, coefficient of variation, minimum and maximum values, skewness, kurtosis, and verification of data normality using an error probability of 5% by the Kolmogorov-Smirnov test. The Pearson correlation between variables was calculated in spreadsheet. The procedure described by Cahn et al. [23] was used to determine the presence of outliers, which in most cases are indicative of errors in reading the values of the variables [24].

The coefficient of variation (CV) of the attributes was rated following Warrick and Nielsen [25], which consider low variability as a CV of <12%, average variability as a CV of 12–60%, and high variability as a CV of >60%.

The spatial dependence was evaluated by setting the semivariogram based on the assumption of stationarity intrinsically estimated by

(1) $$\begin{array}{c} \hat{\gamma }\left(\;h\;\right)=\frac{1}{2N\left(h\right)}\times \overset{N\left(h\right)}{\underset{i=1}{\sum }}{\left[\;Z\left(\;x_{i}+h\;\right)-Z\left(\;x_{i}\;\right)\;\right]}^{2}, \end{array}$$

where $\hat{\gamma }(h)$ is the estimated experimental value and *N*(*h*) is the semivariance number of pairs of measured values *Z*(*x*_*i*_), *Z*(*x*_*i*_ + *h*), separated by a vector *h*.

The software Geoeas [26] was used to generate the semivariogram and choose the best-fit model (exponential, Gaussian, or spherical), as follows.

(i) Exponential model is as follows:
  (2) $$\begin{array}{c} \hat{\gamma }\left(\;h\;\right)=C_{0}+C_{1}{\left[\;1.5\times \frac{h}{A}-0.5{\left(\;\frac{h}{A}\;\right)}^{3}\;\right]}_{0<h<A}. \end{array}$$
(ii) Gaussian model is as follows:
  (3) $$\begin{array}{c} \hat{\gamma }\left(\;h\;\right)=C_{0}+C_{1}{\left[\;1-{e^{-}}^{\left(h^{2}/A^{2}\right)}\;\right]}_{h\neq 0}. \end{array}$$
(iii) Spherical model is as follows:
  (4) $$\begin{array}{c} \hat{\gamma }\left(\;h\;\right)=C_{0}+C_{1}{\left[\;1-{e^{-}}^{\left(h/A\right)}\;\right]}_{h\neq 0}. \end{array}$$

After selecting the model, the semivariogram was subjected to cross-validation process to determine the classification accuracy of the model through software Geoeas [26]. The jack-knifing process [27] was applied for cross-validation, to estimate the variance and the trend of an estimator any. It is based on the removal of one sample of the total range observed and the removed value is recalculated by estimator from the remaining values.

For semivariogram models that showed no defined threshold, indicative of nonstationarity, the trend for the data was withdrawn with the aid of Surfer 7.0 [28].

The trend observed in some variables was removed by polynomial fit (linear, quadratic, or cubic) of the variable and function of coordinates. The residual of this procedure was then used to fit the semivariogram, because according to Vieira et al. [29] the residual becomes the regionalized variable.

## 3. Results and Discussion 

The statistical results for the soil physical properties are presented in Table 2.

The average bulk density was 1.47 g cm^−3^. This result was higher than those of 1.46 g cm^−3^ and 1.29 g cm^−3^ usually found by Camargo et al. [14] and Silva et al. [30] for Alfisol and cohesive Yellow Latosol lands used for cultivating sugarcane in Brazil.

There is still no consensus on the bulk density that is restrictive to the growth of plant roots; several factors influence, for example, culture, the type of soil and the use made of it. In the case of sugar cane Humbert [31] found reduction in the amount of sugarcane roots with soil bulk density of 1.36 g cm^−3^ and a commitment penetration of sugarcane roots in the soil when the density is 1.46 g cm^−3^ or higher in Brazilian soils studies.

The data were normal according to the Kolmogorov-Smirnov test (KS), with kurtosis values close to zero, except for the liquid limit (LL).

There was positive kurtosis for bulk density, soil moisture content, LL, PL, and *σp*10%, which indicates that the data were scattered, and the distribution was narrower than normal (leptokurtic). The other variables had negative kurtosis, where the curve was flatter than normal (platykurtic). The variables showed positive skewness, indicating that most of the data tend towards the minimum values, except *σp*10%, whose asymmetry was negative, indicating that the data tended towards the maximum values found.

There was low variability for bulk density, sand, and LL; average variability for *w*, silt, clay, *σp*19%, and *σp*10%; and high variability for PI, according to the classification by Warrick and Nielsen [25]. Silt and clay could be of higher variability than sand due to their better mobility in the soil.

When comparing this study with other studies that evaluated the preconsolidation tension of Ultisol under the same conditions of soil moisture content and fertigation, the data corroborate Oliveira et al. [4], who found that preconsolidation tension decreased with increasing soil moisture content, which makes the soil more susceptible to compaction at higher soil water content. For the preconsolidation tension (Table 2), one load of 126.28 kPa at a moisture content of 10% or >75.70 kPa for a moisture content of 19% may exceed the carrying capacity of the soil studied and cause plastic deformation, compacting the soil.

There is a direct relationship between the preconsolidation tension and compaction of the soil, where the increase in compression also increases the preconsolidation tension. For this reason, the preconsolidation tension is considered a quantitative indication of the bearing capacity of the soil [6].

Soil conditions of a tension of 126.26 kPa, soil water content of 10%, and fertigation are within the limits observed by other authors [4] (Table 3).

**Figure 2:**
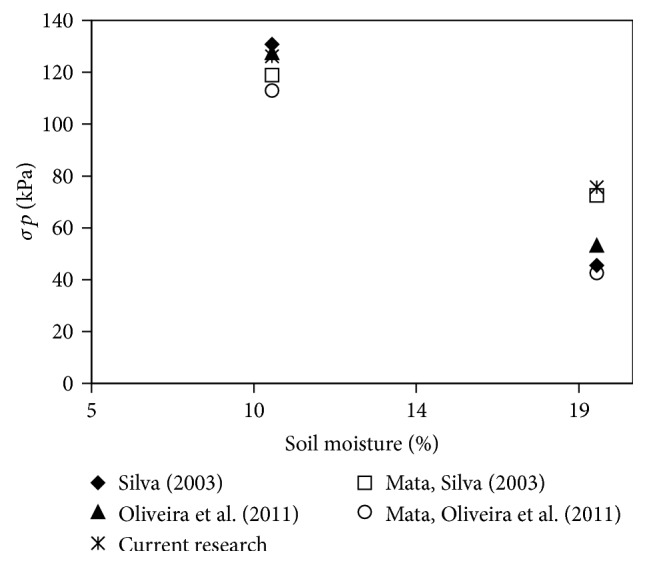
Dispersion of the preconsolidation tension of Ultisol presented in Table 1.

For soil water content of 19%, however, the preconsolidation tension observed was above the pressure encountered by the same authors as preventing fertigation, which may be an indication that additional soil compaction has occurred (Figure 2). As noted, soil moisture content is considered one of the most important factors in the compression process [4] due to the increase in soil strength from the decrease in moisture content. However, when soil moisture content reaches values close to field capacity, the bearing capacity of the soil decreases considerably, increasing the risk of compaction.

According to Alakukku et al. [6], the best strategy for preventing compaction involves the application of external pressures not exceeding the carrying capacity of the soil, which can be estimated by the preconsolidation tension. Likewise, Fritton [10] reviewed research on pedotransfer functions and measures to avoid compaction and concluded that the strain of preconsolidation is appropriate to estimate the tension that can be applied to the soil without causing additional compression.

**Figure 3:**
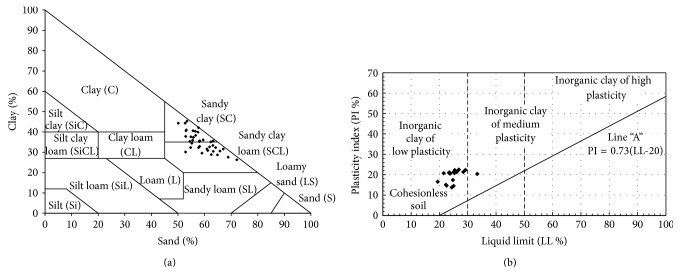
Dispersion of range samples into texture triangle (a) and limits consistency (b).

In Figure 3(a), the soil was distributed across a sandy clay soil and a sandy clay loam soil. From the relationship between the plasticity index (PI) and the liquid limit (LL), clay soil can be classified as inorganic low plasticity (Figure 3(b)). As clay content can increase LL and PI, the low clay may have contributed to the low plasticity index of the soil.

Table 4 shows a positive correlation between density and sand and a significant negative correlation between density and silt, indicating that there is an increase in bulk density with increasing sand content. Both clay and silt and soil moisture content showed positive relationships with the consistency limits LL and PI and the preconsolidation tensions *σp*19% and *σp*10%, which indicate the influence of fine particles and moisture on the soil status.

Through geostatistical analysis, the degree of spatial dependence was classified as strong for *θ*, LL, and *σp*19% and moderate for other variables (Table 5).

All variables showed spatial dependence expressed through the semivariogram models. For clay, *σp*10%, and *σp*19%, the semivariogram was adjusted with the residuals (Table 5). Silt and bulk density were adjusted for a spherical model, and sand and clay were adjusted for a Gaussian model. In Ultisol, Camargo et al. [14] adjusted for a spherical model for bulk density. In Cambisols studied by Rabbi et al. [15], they observed a spherical fit to the bulk density and the sand, silt, and clay.

**Figure 4:**
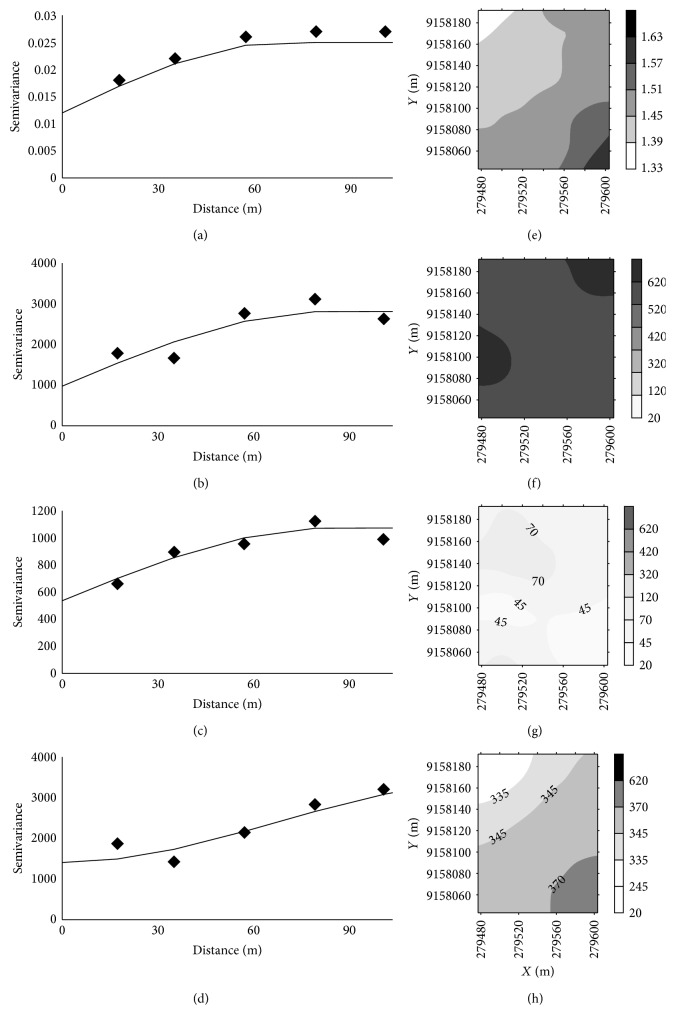
Semivariograms of the parameters of bulk density (a), sand (b), silt (c), and clay (d) and contour maps of bulk density (e), sand (f), silt (g), and clay (h).

**Figure 5:**
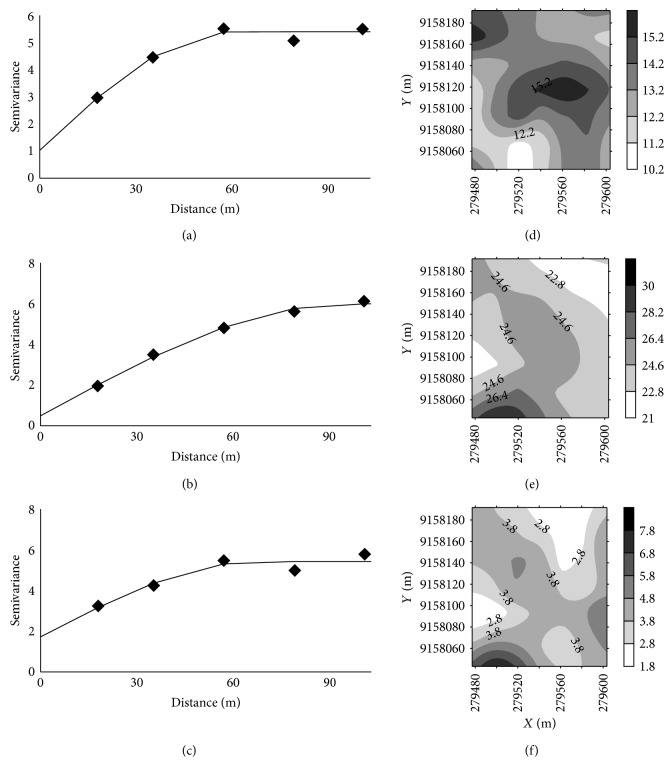
Semivariogram parameters of soil moisture (a), LL (b), and PI (c) and contour maps of soil moisture (d), LL (e), and PI (f).

**Figure 6:**
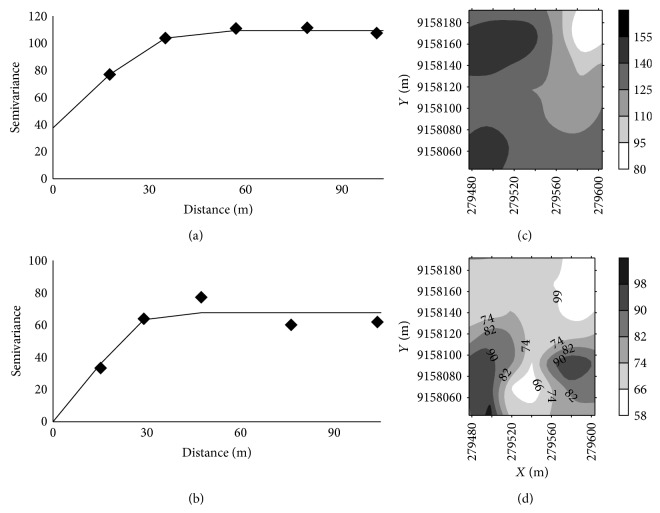
Semivariograms of the parameters of *σp*10% (a) and *σp*19% (b) and contour maps of *σp*10% (c) and *σp*19% (d).

Higher values of range were found for silt, sand, clay, and LL (Figures 4 and 5). The smallest values of range were found for *σp*10%, and *σp*19% (Figure 6). According to Pereira et al. [32], knowledge of the range of spatial dependence allows for the design of future sampling, ensuring the same conditions of the study.

There were also large variations in the nugget effect for soil texture (Figure 4). The nugget effect (*C*_0_) denotes the discontinuity of a phenomenon. According to Vieira et al. [33], high values of the nugget effect indicate variability not detected by the sampling process.

**Figure 7:**
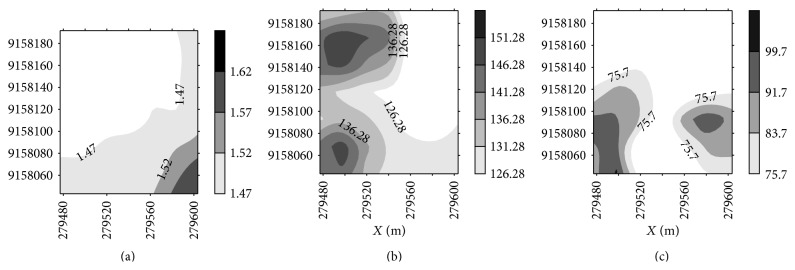
Contour maps of the bulk density from 1.47 g cm^−3^ (a); preconsolidation tension with soil water content of 10% for tension equal to or greater than 126.28 kPa (b); and preconsolidation tension with soil water content of 19% for tension equal to or greater than 75.70 kPa (c).

In order to refine the map of soil density and preconsolidation tension, a new contour map is shown in Figure 7 separating the areas with densities above 1.47 g cm^−3^ and tension above the average observed in the present study. Based on the average of all the values observed for the bulk density 1.47 g cm^−3^, it was decided to refine the map to check which areas have values equal to or above 1.47 g cm^−3^. The same procedure was performed with the mean value of the preconsolidation pressure, taking as reference the average value of 126.28 kPa for moisture 10% and the average value of 75.70 kPa for moisture 19%. In these maps, we can observe that half of the area lying above the bulk density of 1.47 g cm^−3^; that is, more than half of public areas have values above what is normally observed in other studies. More than half of the area when soil water content is 10% can support tensions of 126.28 kPa, which is a value occurring within those verified by other authors' standards. The tension with soil water content of 19% (Figure 7(c)) can define locations where tension has already been introduced above what is reported in the literature. In this case, the maps in Figures 7(b) and 7(c) indicate the areas that can support heavier equipment (dark areas) and that tolerate lighter vehicles only (light areas) without causing further compaction.

**Figure 8:**
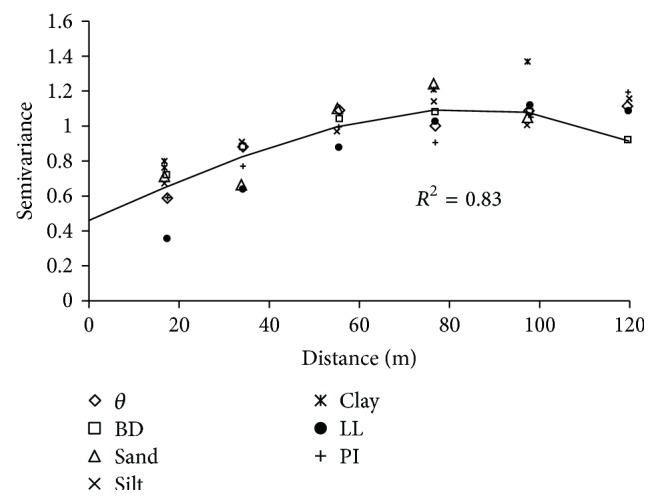
Scaled semivariogram and adjusted spherical model to BD, *θ*, sand, silt, clay, LL, and PI.

To verify that the semivariograms exhibit the same spatial variability structure, a scaled semivariogram was built as in Vieira et al. [33]. From Figure 8, it can be seen that the scaled variables BD, *θ*, sand, silt, clay, LL, and PI showed similar structures of dependence, adjusting the spherical model with an *R* ^2^ of 82%, as shown in Figure 8. There was an increasing curve until distance of 80 m stabilizes after this distance. For this technique, *σp*10% and *σp*19% were not included in the figure because it did not present the same structure and, therefore, the model found would not be able to adequately describe these two variables. The stability of attributes and semivariogram (Figure 8), confirming that stationarity of data, because the data as a whole also showed semivariograms well defined and without tendency [34].

## 4. Conclusions

The results show that half of the area had a bulk density above 1.47 g cm^−3^. Tillage in soil moisture content around 19% should be avoided or should be chosen a management of soil with lighter vehicles in this moisture content. The geostatistical methods allowed the identification of the spatial dependence for all variables. Computational effort can be saved through using single semivariogram scaled variables representing the BD, *θ*, sand, silt, clay, LL, and PI variables. The generated maps of preconsolidation tension can indicate areas that support heavier equipment and areas that can tolerate only lighter vehicles without causing further compaction.

## Figures and Tables

**Table 1:** Physical characterization of Ultisol used for growing sugarcane by Bebé et al., 2009 [1].

Bulk density	Sand	Silt	Clay	FC	PWP
(gcm^−3^)	(%)	(%)	(%)	(%)	(%)
1.47	58.77	6.06	35.15	18.8	11.58

FC: field capacity; PWP: permanent wilting point.

**Table 2:** Statistical attributes for the parameters under study.

	BD	*θ*	Sand	Silt	Clay	LL	PI	*σp*10%	*σp*19%
Number	49.00	48.00	39.00	39.00	39.00	46.00	44.00	45.00	49.00
Mean	1.47	13.42	58.77	6.06	35.15	24.24	3.86	126.28	75.70
Median	1.49	13.42	58.02	6.20	35.32	23.84	3.74	130.45	73.30
Minimum	1.17	7.73	50.12	0.17	26.32	20.33	0.37	70.50	61.50
Maximum	1.89	18.15	72.12	17.02	45.32	33.00	11.74	157.80	95.00
Variance	45.21	5.06	1261.14	4723.50	192.74	5.47	5.51	617.30	147.91
Skewness	0.23	0.03	0.64	0.76	0.34	1.15	0.98	−0.91	0.50
Kurtosis	0.09	0.11	−0.24	−0.60	−0.46	3.29	1.75	0.19	−1.21
SD	0.16	2.25	50.07	35.81	48.39	2.34	2.35	24.85	12.16
CV	11	9	17	59	14	10	61	20	16
KS	0.08	0.05	0.14	0.10	0.09	0.09	0.06	0.15	0.17

BD: Bulk density (g·cm^−3^); *θ*: soil moisture content (g·g^−1^); Sand, Silt and Clay: (%); LL: liquid limit (%); PI: plasticity index (%); *σp*: pre-consolidation tension (kPa); SD: standard deviation; CV: coefficient of variation; KS: test of normality of Kolmogorov-Smirnov.

**Table 3:** Preconsolidation tension (kPa) of Ultisol under different managements and soil moisture content.

Author		Soil water content (*θ*, %)	
		*σp*10%	*σp*19%
Silva (2003) [12]	Fertigation	130.8	45.5
	Forest	119	72.5

Oliveira et al. (2011) [4]	Fertigation	127.5	53.3
	Forest	113	42.67

*σp*: pre-consolidation tension (kPa).

**Table 4:** Matrix linear correlation between the physical and mechanical soil attributes.

	BD	*θ*	Sand	Silt	Clay	LL	PI	*σp*10%	*σp*19%
BD	1.00	−0.10	0.44	−0.38	−0.17	−0.42	−0.46	−0.26	0.00
*θ*		1.00	−0.49	0.05	0.47	0.41	0.10	0.03	−0.05
Sand			1.00	−0.40	−0.74	−0.69	−0.46	−0.40	−0.05
Silt				1.00	−0.32	0.39	0.29	0.25	−0.12
Clay					1.00	0.42	0.27	0.26	0.14
LL						1.00	0.66	0.44	0.27
PI							1.00	0.26	0.24
*σp*10%								1.00	0.48
*σp*19%									1.00

BD: Bulk density (g·cm^−3^); *θ*: soil moisture content (g·g^−1^); Sand, Silt and Clay: (%); LL: liquid limit (%); PI: plasticity index (%); *σp*: pre-consolidation tension (kPa).

**Table 5:** Fitting parameters of the experimental semivariogram.

	BD	*θ*	Sand	Silt	Clay	LL	PI	*σp*10%	*σp*19%
*C*_0_^#^	0.012	1.03	1358.54	536.15	1407.73	0.48	1.72	37.41	0.00083
*C*_1_^##^	0.014	4.36	1690.21	536.51	2418.1	5.51	3.71	72.01	67.61
*a* ^###^	66.96	57.37	48.84	80.65	89.56	92.7	65.46	44.64	18.34
Model	Spherical	Spherical	Gaussian	Spherical	Gaussian^*∗*^	Spherical	Spherical	Spherical^*∗*^	Gaussian^*∗*^
SDD^†^ (%)	48	19.10	44.56	49.98	36.8	8.01	31.67	34.18	0.001
Mean	−0.02	−0.009	−0.004	−0.013	0.004	−0.031	−0.025	−0.007	−0.015
SD^††^	1.107	1.188	0.949	1.112	0.106	1.065	1.106	0.832	1.192
*R* ^2^^†††^	0.89	0.98	0.86	0.93	0.92	0.99	0.95	0.97	0.90

^#^Nugget effect, ^##^structured variance, ^###^range, ^†^spatial dependency degree = [*C*_0_/(*C*_0_ + *C*_1_)] × 100, ^††^standard deviation, ^†††^coefficient of determination, and ^*∗*^semivariogram of the residual.
